# Geriatric risk and protective factors for serious COVID-19 outcomes among older adults in Shanghai Omicron wave

**DOI:** 10.1080/22221751.2022.2109517

**Published:** 2022-08-31

**Authors:** Guanzhu Lu, Yi Zhang, Haocheng Zhang, Jingwen Ai, Liu He, Xiaoling Yuan, Suxia Bao, Xiaohua Chen, Hongyu Wang, Jianpeng Cai, Sen Wang, Wenhong Zhang, Jie Xu

**Affiliations:** a Department of Infectious Disease, Shanghai Ninth People’s Hospital, Shanghai Jiao Tong University School of Medicine, Shanghai, People’s Republic of China; b Department of Infectious Diseases, National Medical Center for Infectious Diseases, Shanghai Key Laboratory of Infectious Diseases and Biosafety Emergency Response, Huashan Hospital, Fudan University, Shanghai, People’s Republic of China; c Huashen Institute of Microbes and Infections, Shanghai, People’s Republic of China; d Medical section, Shanghai Ninth People’s Hospital, Shanghai Jiao Tong University School of Medicine, Shanghai, People’s Republic of China; e Department of Infectious Disease, Shanghai Sixth People’s Hospital, Shanghai Jiao Tong University School of Medicine, Shanghai, People’s Republic of China

**Keywords:** COVID-19, Omicron, elderly, risk factor, vaccination

## Abstract

Shanghai has been experiencing the Omicron wave since March 2022. Though several studies have evaluated the risk factors of severe infections, the analyses of BA.2 infection risk and protective factors among geriatric people were much limited. This multicentre cohort study described clinical characteristics, and assessed risk and protective factors for geriatric Omicron severe infections. A total of 1377 patients older than 60 were enrolled, with 75.96% having comorbidities. The median viral shedding time and hospitalization time were nine and eight days, respectively. Severe and critical were associated with longer virus clearance time (aOR [95%CI]:0.706 (0.533–0.935), *P* = .015), while fully vaccinated/booster and paxlovid use shortened viral shedding time (1.229 [1.076–1.402], *P* = .002; 1.140 [0.019–1.274], *P* = .022, respectively). Older age (>80), cerebrovascular disease, and chronic kidney disease were risk factors of severe/critical. Fully vaccination was a significant protective factor against severe infections (0.237 [0.071–0.793], *P* = .019). We found patients with more than two comorbidities were more likely to get serious outcomes. These findings demonstrated that in the elderly older than 60 years old, older age (aged over 80), cerebrovascular disease, and chronic kidney disease were risk factors for severe infection. Patients with more than two comorbidities were more likely to get serious outcomes. Fully vaccinated/booster patients were less likely to be severe and vaccinations could shorten viral shedding time. The limitation of lacking an overall spectrum of COVID-19 infections among elders could be compensated in other larger-scale studies in the future.

## Introduction

The COVID-19 pandemic, caused by severe acute respiratory syndrome coronavirus 2 (SARS-CoV-2), has caused 0.53 billion COVID-19 infections and 6.3 million deaths by 12 June 2022 [1]. The Omicron variant is the most recent variant of concern since its first detections in Botswana and South Africa in November 2021 and has occupied 34% of all strains by 28 May, 2022 [2]. The Omicron variant was featured with high transmissibility and striking antibody evasion, including several subvariants, BA.1, BA.2, BA,3, BA.4, and BA.5 [3–5]. Though Omicron was reported to have a lower mortality rate and severity rate, it led to more than 9000 deaths in Hongkong [6].

During the global Omicron wave, older adults have been identified by several studies to be disproportionately affected by COVID-19. Patients older than 60 were considered in a high-risk category for serious illness from the COVID-19 according to the national guidance. CDC released the COVID-19 guidance and revealed patients aged between 50 and 64 were 4.3 times at risk of progressing to severe status compared to 18–39 years, and the risk ratio even reached 10.6 in patients older than 85 [7]. Over 95% of fatal cases in Hongkong during the Omicron pandemic were senior citizens aged 60 or older. Previous risk analyses also showed that patients with certain medical conditions could be more likely to have severe illnesses, such as cancer, chronic kidney disease, chronic lung diseases, diabetes, heart conditions, etc. [8]. For geriatric patients, chronic kidney disease and previous stroke obviously contributed to fatality in hospitalized elderly patients [9].

In the fifth COVID-19 wave in Hongkong during early 2022, the exceedingly high death rate was attributed to the unvaccinated/not fully elderly [10]. The case fatality rate of unvaccinated people was 3.04%, while the fatality rate was only 0.17% and 0.04% in patients who received two-dose and three-dose vaccination, respectively. During COVID-19 infections, cytokines IL2R, IL6, IL8, IL10, and TNF-αcould elevate in severe patients indicating pulmonary inflammation and proinflammatory cytokines, which indicated that inflammatory cytokines are associated with disease severity [11,12].

Shanghai has been undergoing an Omicron wave since March 2022. The effective reproduction number of the BA.2 subvariant was 1.4-fold higher than that of BA.1 [13], which poses a great threat to COVID-19 prevention and control. Though several studies have evaluated the risk factors of severity or death [14–17], the analyses of BA.2 infection risk and protective factors among geriatric people were restricted. As a result, there is a pressing need to determine the effect of Omicron infection in older adults and identify the factors in disease progression and prevention. Thus, we here conducted this multicentre cohort study on the geriatric population in the Shanghai Omicron wave from March 2022 to May 2022.

## Methods

### Study design and patient enrollment

We conducted this multicentre cohort study in Shanghai ninth people’s hospital, Huashan Hospital, and Shanghai sixth people’s hospital between April 2022 and May 2022. We enrolled patients who had confirmed COVID-19 by SARS-CoV-2 real-time polymerase reaction chain (RT–PCR) tests and were aged over 60 years admitted to the designated hospitals, which admitted mostly COVID-19 patients with high-risk factors or with comorbidities. This study was approved by the ethical committee of Shanghai ninth people’s hospital (SH9H-2022-T139-1), Shanghai sixth people’s hospital (2022KY-069), and Huashan Hospital (KY2022-596). Patients without consent or complete medical history were excluded from this study.

The clinical course classification and treatment was according to the ninth version of the national COVID-19 guidance. Participants were considered to have severe Omicron infection when SpO_2_ ≤93% in the resting state. The criteria for critical infections included at least one of the following: (1) respiratory failure requiring mechanical ventilation, (2) shock, and (3) a combination of other organ failures requiring ICU care.

Nine types of comorbidities were confirmed in this study, including hypertension, diabetes, cardiovascular disease (including coronary heart disease, atrial fibrillation, pulmonary embolism, rheumatic heart disease, old myocardial infarction, and cardiac insufficiency), cerebrovascular disease (cerebral infarct and hemorrhage), tumour, respiratory disease (bronchiectasis, bronchitis, chronic obstructive pulmonary disease, emphysema, history of tuberculosis and asthma), rheumatic immune disease (systemic lupus erythematosus, rheumatoid arthritis, scleroderma, and autoimmune thrombocytopenia), and hepatic disease (fatty liver, hepatitis B, and liver cirrhosis). Chronic kidney disease (CKD) included chronic renal failure, chronic nephritis, and diabetic nephropathy, among which 12 out of 49 patients were evaluated as CKD stage 5. We then classified these patients into comorbidity ≤1 and comorbidity ≥2 according to comorbidity numbers.

Participants received oropharynx swab SARS-CoV-2 RT–PCR test using SARS-CoV-2 ZC-HX-201-2 kit (Biogerm, Shanghai, China) during hospitalization once a day, and will be considered as virus clearance when two consecutive negative nucleic acids of SARS-CoV-2 RT–PCR test were reported (cycle threshold value large than 35 in both ORF1ab and N genes), tested at intervals of at least 24 h. Viral shedding time was defined as the first day of the positive nucleic acid test to the date of the first negative test of the consecutive negative results. The serum cytokine concentrations of IL-1β, IL2R, IL6, IL8, IL10, and TNF-α were measured using blood samples sent to the hospital clinical laboratory, and the cut-off values were determined by the clinical laboratory. Absolute cell numbers of peripheral lymphocyte counts were recorded as well.

We recorded the baseline information, comorbidities, clinical manifestations, prognosis, and laboratory examinations, including epidemiologic information simultaneously and performed risk analysis for severe progression among these enrolled patients.

### Statistical analyses

Continuous variables were described as mean values ± standardized deviation if they were Gaussian distribution by the Kolmogorov–Smirnov test. Otherwise, they were expressed as median and interquartile ranges. Pearson’s Chi-Square test was used to evaluate independent binomial variables. We evaluated the factors influencing viral clearance using Cox regression and calculated adjusted hazard ratio (aHR) and 95% confidence interval (95%CI), covariates including age, clinical diagnosis, viral pneumonia on CT, comorbidity or not, vaccination status, and Paxlovid use condition. Logistic regression was employed to analyze risk factors for severe/critical patients, adjusted odds ratio (aOR and 95%CI were calculated), and covariates including sex, age, different comorbidities, vaccination status, and viral pneumonia on CT. *P*-values <.05 were considered statistically significant. The receiver operating characteristic (ROC) curve was used to illustrate the diagnostic ability of lymphocyte, CD4, and CD8 counts in distinguishing severe patients. Statistical analysis was performed using SPSS (v 23.0) software and figures were generated using GraphPad Prism (v 8) and RStudio (v 1.2).

## Results

### Clinical manifestations of enrolled patients

A total of 1781 patients were diagnosed with COVID-19 in the period under study and 1377 were eligible in our analysis, of which we excluded 401 patients younger than 60 years old, and three had an unclear medical history. All basic characteristics and laboratory examinations are listed in Table 1. The median age for participants was 76 years old (IQR: 69–84), and the proportion of patients aged 61–70, 71–80, and over 80 years old was 30.57% (421/1377), 33.33% (459/1377), and 36.09% (497/1377), respectively. Unvaccinated patients accounted for the most among enrolled patients (71.25%, 988/1377), followed by patients who had received the third booster dose vaccination (15.61%, 215/1377) and two-dose vaccination (10.68%, 147/1377).

**Table 1.** The basic characteristics of enrolled patients.

*N*	1377
Age, median (IQR)	76 (69–84)
61–70 years (*N*, %)	421 (30.57%)
71–80 years (*N*, %)	459 (33.33%)
>80 years (*N*, %)	497 (36.09%)
Gender, *N* (%)	
Male	642 (46.62%)
Vaccination, N (%)	
Unvaccinated	988 (71.75%)
One dose	14 (1.02%)
Two doses	147 (10.68%)
Three doses	215 (15.61%)
Unclear	13 (0.94%)
Viral Pneumonia on CT, *N* (%)	224 (16.27%)
Symptoms, *N* (%)	
Fever	273 (19.83%)
Sore throat	247 (17.94%)
Cough	587 (42.63%)
Diarrhoea	4 (0.29%)
Nasal obstruction or rhinorrhoea	145 (10.53%)
Impaired sense of smell	2 (0.15%)
Treatment, *N* (%)	
Respiratory support	161 (11.69%)
Dialysis	6 (0.44%)
Antiviral drug	553 (40.16%)
Glucocorticoid	120 (8.71%)
Hospitalization days, median (IQR)	8 (5–11)
Viral shedding time, median (IQR)	9 (6–12)
Outcome, *N* (%)	
Death	7 (0.51%)
Relieve	1370 (99.49%)
Laboratory examinations	
WBC <3.0*10^9/L	51/1212 (4.21%)
Lymphocyte <1*10^9/L	193/1095 (17.63%)
ALT >40 U/L	152/1175 (12.94%)
AST >35 U/L	253/1175 (21.53%)
D-dimer >0.5 mg/L	545/1067 (51.08%)
CRP >10 mg/L	337/1209 (27.87%)
IL-1β >5 pg/ml	111/579 (19.17%)
IL-2R >710 U/ml	167/514 (32.49%)
IL-6 >3.4 pg/ml	437/573 (76.27%)
IL-10 >9.1 pg/ml	34/503 (6.76%)
LDH >250 U/L	239/759 (38.60%)
BNP >100 pg/mL	244/818 (29.83%)

WBC: white blood cell; ALT: alanine aminotransferase; AST: Aspartate Transaminase; CRP: C-reactive protein; IL: interleukin; LDH: lactate dehydrogenase; BNP: N-terminal pro-B-type natriuretic peptide.

The cut-offs for laboratory examinations: WBC: 3.0*10^9/L; Lymphocyte: 1*10^9/L; ALT: 40 U/L; AST: 35 U/L; D-dimer: 0.5 mg/L; CRP: 10 mg/L; IL-1β: 5 pg/ml; IL-2R: 710 U/ml; IL-6 >3.4 pg/ml; IL-10: 9.1 pg/ml; LDH: 250 U/L; BNP: 100 pg/ml.

The symptoms at admission included cough (587, 42.63%), fever (273, 19.83%), sore throat (247, 17.94%), nasal obstruction or rhinorrhea (142, 10.53%), diarrhea (4, 0.29%), impaired sense of smell (2, 0.15%), etc. (Table 1). Only 16.27% of patients had abnormal chest computed tomography (CT) images considering viral pneumonia. We next analyzed the baseline laboratory examinations at admission. The lymphocyte less than 1 × 10^9/L was found in 17.63% of patients. More than half of the patients owned an elevated D-dimer and interleukin 6 (IL-6) level. Besides, 38.60% and 29.83% of patients had an abnormal lactate dehydrogenase (LDH) and N-terminal pro-B-type natriuretic peptide level (NT-proBNP).

The median hospitalization day was 8 (IQR: 5–11) days. 99.13% (1365/1377) patients were discharged, and 7 (0.51%) patients died. The patients died of acute myocardial infarction (*n* = 1), brain stem infarction (*n* = 1), aspiration pneumonia (*n* = 3), bacterial pneumonia (*n* = 1), and biliary tract infection-induced septic shock (*n* = 1).

As shown in Supplementary Figure 1, among all the diseases, hypertension was the most found (54.97%), followed by diabetes (23.53%), cardiovascular disease (20.70%), cerebrovascular disease (14.16%), and tumour (10.17%). Of all participants, 75.96% had at least one comorbidity, while 24.04% had no comorbidity.

### Risk and protective factors of virus clearance

**Figure 1.**
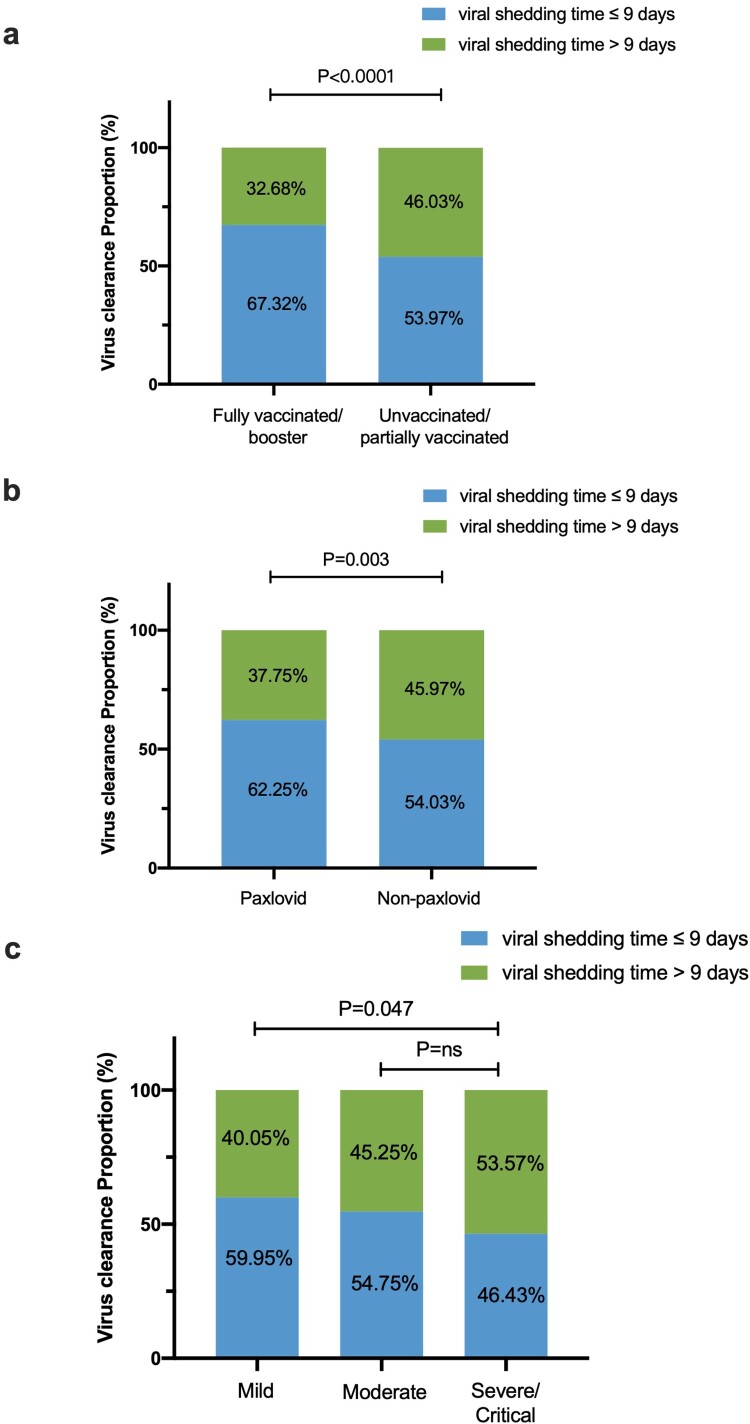
The proportion of viral shedding time ≤9 days and >9 days according to clinical diagnosis, vaccination, and paxlovid use. (a) The proportion of viral shedding time ≤9 days and >9 days according to clinical diagnosis. (b) The proportion of viral shedding time ≤9 days and >9 days according to paxlovid use. (c) The proportion of viral shedding time ≤9 days and >9 days according to vaccination.

We included 1337 patients with clear viral shedding time (VST) in the analysis. The median viral shedding time of participants was nine days (IQR: 6–11 days) (Table 1). Patients in the fully vaccinated/booster group, the paxlovid group, and the mild group were more likely to clear the virus within nine days (67.32% vs. 53.97%, *P* < .0001; 62.25% vs. 54.03%, *P* = .003; 59.95% vs. 46.43%, *P* = .047), as shown in Figure 1.

Through multivariate analyses, the VST was longer in the severe/critical group than in the non-severe/critical group (adjusted HR, 0.706 [95%CI: 0.533–0.935]; *P* = .015). Compared to unvaccinated/partially vaccinated patients, two-dose or three-dose vaccination significantly shortened the VST (adjusted HR, 1.229 [95%CI: 1.076–1.402]; *P* = .002). Paxlovid could also shorten the VST (adjusted HR 1.145 [95%CI: 1.024–1.281]; *P* = .022) (Table 2 and Figure 1).

**Table 2.** Influencing factors of viral shedding time among geriatric patients.

	Univariate		Multivariate	
	HR	*P*	Adjusted HR	*P*
Age groups				
Age <70	1.00		1.00	
70–80	0.922 (0.807–1.054)	.234	0.963 (0.839–1.106)	.596
>80	**0.829 (0.726–0.946)**	**.006**	0.890 (0.771–1.028)	.112
Clinical diagnosis				
Mild	1.00		1.00	
Moderate	0.931 (0.832–1.041)	.21	0.946 (0.829–1.080)	.412
Severe/critical	**0.679 (0.516–0.893)**	**.006**	**0.706 (0.533–0.935)**	**.015**
Viral Pneumonia on CT	0.975 (0.844–1.127)	.733	1.010 (0.85301.196)	.907
Comorbidity	0.931 (0.821–1.057)	.268	0.706 (0.533–0.935)	.508
Vaccination status				
Unvaccinated/partially	1.00		1.00	
Fully vaccinated/booster doses	**1.293 (1.144–1.461)**	**<.001**	**1.229 (1.076–1.402)**	**.002**
Paxlovid	1.100 (0.986–1.227)	.089	**1.140 (1.019–1.274)**	**.022**

Cox regression was used in the analysis, covariates included age, clinical diagnosis, viral pneumonia on CT, comorbidity or not, vaccination status, and paxlovid use. For vaccination status, unvaccinated/partial vaccinated was used as the reference group. For age groups, age <70 was used as the reference group. For clinical diagnosis, mild was used as the reference group.

### Risk and protective factors of disease progression to severe/critical state

We next analyzed influencing factors of enrolled severe/critical status patients (*n* = 61). The univariate analyses showed patients >80 years old, combined with cerebrovascular disease, cardiovascular disease, chronic kidney disease, and respiratory diseases were more likely to have disease progression (Table 3**)**. By multivariate analyses, we identified age >80-year-old patients were more likely to become severe/critical than those <70 years old (adjusted OR, 3.071 [95%CI: 1.352–6.976]; *P* = .007). The adjusted odds ratio for cerebrovascular disease and chronic kidney disease was 3.275 (95%CI: 1.814–5.913; *P* < .001) and 3.978 (95%CI: 1.595–9.922; *P* = .003). Compared to unvaccinated/partially vaccinated patients, fully vaccinated/booster patients had an aOR of 0.237 (95%CI: 0.071–0.793; *P* = 0.019), an obvious protective factor of severe/critical outcome.

**Table 3.** Risk factors of progressing to severe/critical (*n* = 61) among geriatric patients.

	Univariate		Multivariate	
	OR	*P*	Adjusted OR	*P*
Female	0.682 (0.407–1.143)	.147		
Age groups				
Age <70	1.00		**1.00**	
70–80	1.674 (0.674–3.911)	.279	1.504 (0.611–3.702)	.375
>80	**4.396 (2.031–9.516)**	**<.001**	**3.071 (1.352–6.976)**	**.007**
Tumour	0.609 (0.218–1.704)	.345	0.769 (0.264–2.241)	.63
Hypertension	0.964 (0.576–1.613)	.888	0.659 (0.369–1.177)	.159
Diabetes	1.164 (0.649–2.088)	.611	0.979 (0.517–1.856)	.949
Cerebrovascular disease	**4.026 (2.341–6.922)**	**<.001**	**3.275 (1.814–5.913)**	**<.001**
Cardiovascular disease	**0.560 (0.320–0.979)**	**.042**	1.481 (0.805–2.275)	.207
Chronic kidney disease	**3.932 (1.689–9.156)**	**.001**	**3.978 (1.595–9.922)**	.003
Respiratory diseases	**2.128 (1.019–4.444)**	**.045**	1.477 (0.669–3.263)	.335
Hepatic disease	2.420 (0.302–19.415)	.405	5.767 (0.661–50.294)	.113
Vaccination status				
Unvaccinated/partial vaccinated	1.00		1.00	
Fully vaccinated/booster	**0.135 (0.042–0.435)**	**<.001**	**0.237 (0.071–0.793)**	**.019**
Viral Pneumonia on CT	1.010 (0.505–2.020)	.978	0.911 (0.443–1.873)	.800

Logistic regression was used, covariates included sex, age, different comorbidities, vaccination status, and viral pneumonia on CT. For vaccination status, unvaccinated/partial vaccinated was used as the reference group. For age groups, age <70 was used as the reference group.

Furthermore, we classified patients into comorbidity ≤1, and comorbidity ≥2 groups according to their chronic medical conditions. Compared to the comorbidity ≤1 group, the comorbidity ≥2 group was two times more likely to have severe/critical outcomes (adjusted OR, 2.068 [95%CI: 1.210–3.536], *P* = .008) (Table 4). What’s more, unvaccinated/partially vaccinated patients with more than two comorbidities had a higher risk in geriatric populations in all older age groups. The risk of severe/critical disease progression of unvaccinated/partially vaccinated with more than 2 comorbidities was 5.357 (95%CI: 1.258–22.820), 3.196 (1.088–9.392), and 1.992 (1.025–3.871) times higher in 60–70-year-olds, 70–80-year-olds, and over 80-year-old groups (Supplementary Table 1).

**Table 4.** Risk factors of progressing to severe/critical among geriatric patients using comorbidity numbers.

	Univariate		Multivariate	
	OR	*P*	Adjusted OR	*P*
Female	0.682 (0.407–1.143)	.147	0.651 (0.384–1.102)	.11
Age <70	1.00		**1.00**	
70–80	1.674 (0.674–3.911)	.279	1.485 (0.612–3.605)	.382
>80	**4.396 (2.031–9.516)**	**<.001**	**3.193 (1.449–7.036)**	**.004**
Comorbidities ≥2	**2.389 (1.407–4.056)**	**.001**	**2.068 (1.210–3.536)**	**.008**
Unvaccinated/partial vaccinated	1.00		1.00	
Fully vaccinated/booster	**0.135 (0.042–0.435)**	**<.001**	**0.214 (0.065–0.705)**	**.011**
Viral Pneumonia on CT	1.010 (0.505–2.020)	.978	0.886 (0.438–1.793)	.737

Logistic regression was used, and covariates included sex, age, comorbidity group, vaccination status, and viral pneumonia on CT. For vaccination status, unvaccinated/partial vaccinated was used as the reference group. For age groups, age <70 was used as the reference group.

**Figure 2.**
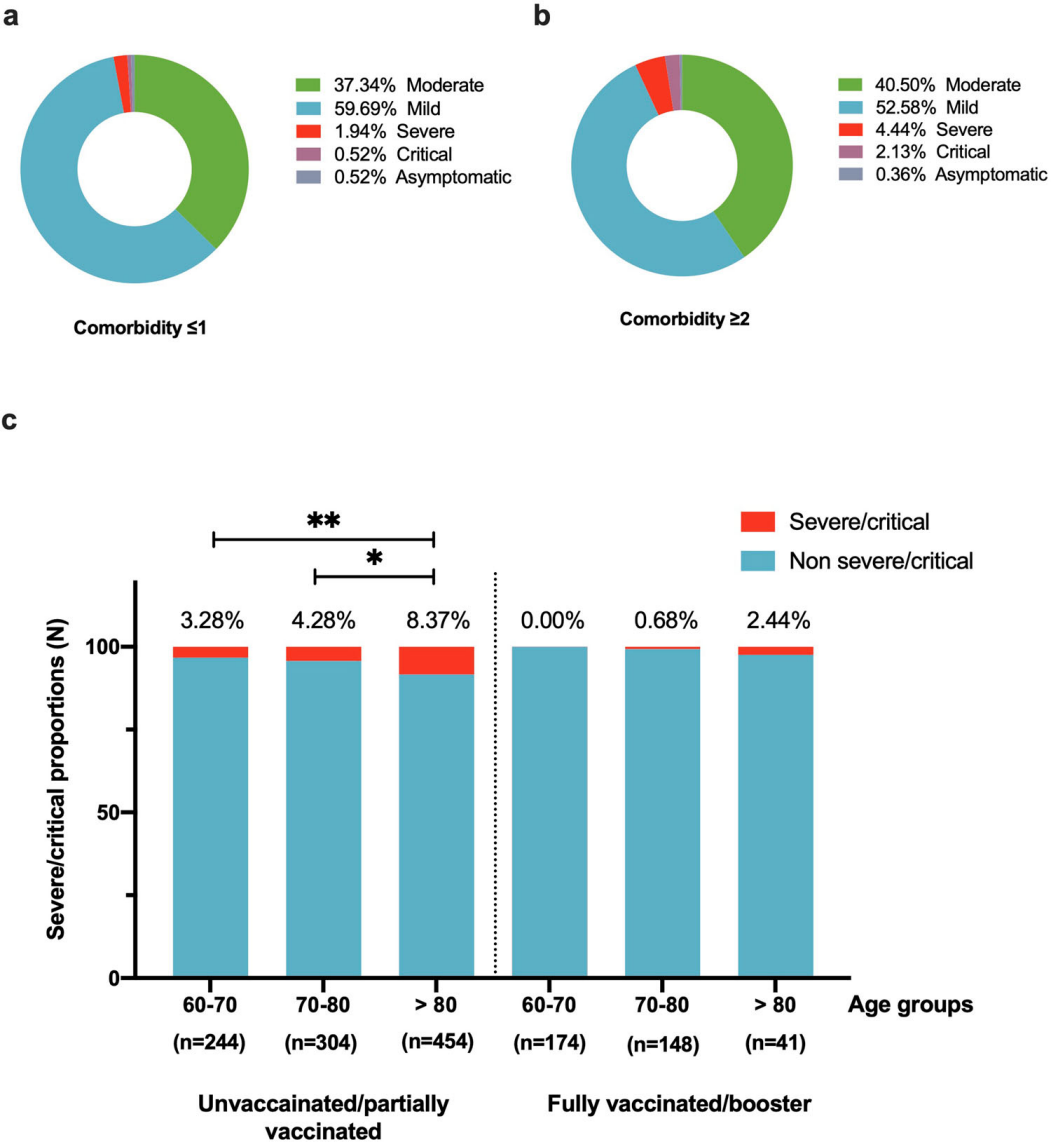
The severe/critical rate according to comorbidity numbers, vaccination, and age. (a) The severe/critical rate in patients with comorbidities ≤1 and comorbidities ≥2. (b) The severe/critical rate in patients in different age groups with different vaccination doses.

We further evaluated vaccine status against COVID-19 severity in different older age groups (Figure 2). In the unvaccinated group, the severe/critical rate of patients aged >80 was significantly higher than 60–70 years old and 70–80 years old (8.37% vs. 3.28%, *P* = .010; 8.37% vs. 4.28%, *P* = .027). For participants who received fully/booster vaccination, the severe/critical rate was 2.44% in patients older than the 80-year-old group, relatively lower than those unvaccinated/partially vaccinated (2.4% vs. 8.37%, *P* = .235).

### Differential laboratory examinations and cytokines between severe and non-severe patients

**Figure 3.**
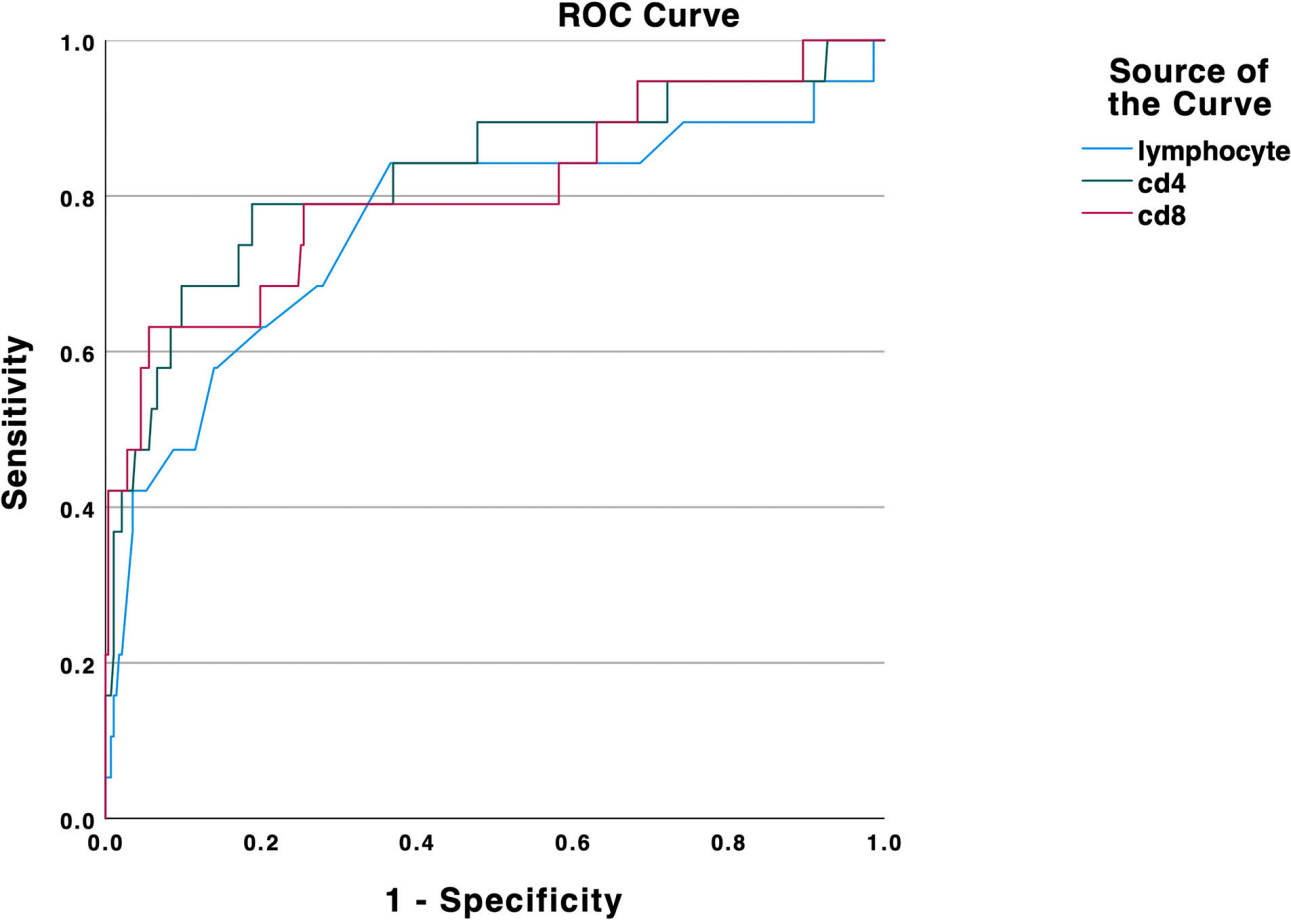
Laboratory markers of severe/critical patients. The indicators include lymphocyte numbers, CRP, BNP, LDH, D-dimer, and cytokines (IL2R, IL6, IL8, IL10, TNF-α) and numbers of CD4^+^ and CD8^+^ cells.

**Figure 4.**
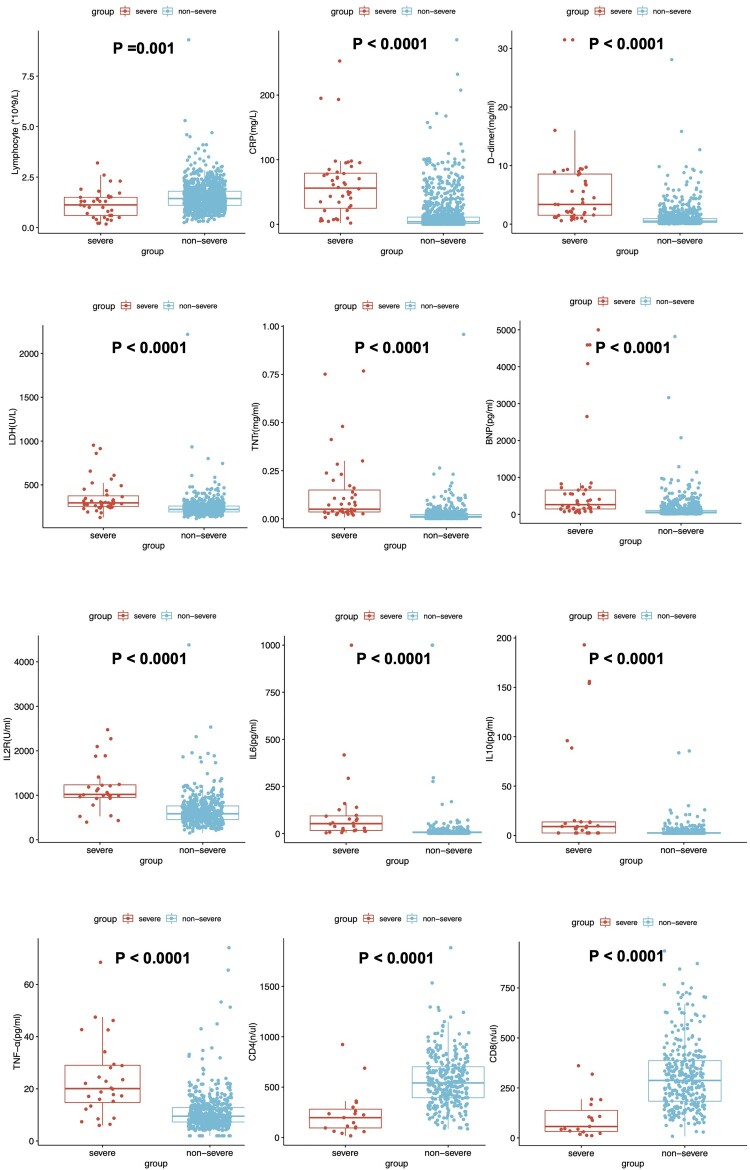
ROC curve of lymphocyte, CD4, and CD8 in distinguishing severe/critical.

We found an increased level of CRP, LDH, D-dimer, Troponin T (TNT), and NT-proBNP in the severe group. Additionally, a decreased level of lymphocytes was found in severe patients. The inflammatory factors, such as IL-2R, IL-6, IL-10, and TNF-α, were significantly increased in the severe/critical patients group (Figure 3). The CD4^+^T cells and CD8^+^T cell counts were lower than those of the non-severe group. We then depicted ROC curves of lymphocytes, CD4, and CD8, and found that they had a satisfying ability of differentiating between severe and non-severe patients with area under the curve of 0.765, 0.829, and 0.806, respectively (Figure 4).

## Discussion

This multicentre study was conducted among older adults infected with the Omicron variant. We found fully vaccination/booster is a significant protective factor against severe/critical infection, and we identified several risk factors for severe disease progression, including aged more than 80, chronic kidney disease, and cerebrovascular disease. Aged more than 80, and severe/critical state could lengthen patients’ viral shedding time, while the paxlovid use could accelerate virus clearance. These findings could help further refine the “high-risk population” among old adults, and alarm the physicians to provide timely clinical care and treatment.

In China, due to the adoption of the prevention and treatment strategy of “receive as much as possible” and “as-received treatment”, most patients can be admitted to centralized isolation sites or designated hospitals. The elderly population screened in this study are all “vulnerable groups”. Timely diagnosis of this group of people and timely admission and medical care prescribed for them can further reduce the critical progression rate of vulnerable groups.

Our study found specific risk factors in geriatric patients for severe COVID-19, including older age (>80), cerebrovascular disease, and chronic kidney disease, which indicates that more attention should be paid to these patients in clinical settings. Moreover, patients from the nursing home were highly vulnerable to serious outcomes of COVID-19 [14]. Additionally, male gender, dyspnoea, and dementia were reported to be associated with a greater risk of death in the elderly from COVID-19 infection [17]. We also illustrated that elderly patients with more than two comorbidities were more likely to get severe/critical outcomes, requiring careful observation. The frequent occurrence of multiple comorbidities has been revealed [14] to have an increased mortality risk according to our study.

Fully vaccinated/booster was a significant protective factor in all age groups of the elderly, the vaccine effectiveness against severe was approximately 76%. Although geriatric patients were vulnerable to being infected by SARS-CoV-2, especially with underlying comorbidities, fully vaccination/booster vaccination can offer substantial protection against COVID-19. Generally, the relative risk of dying from COVID-19 among unvaccinated persons was 33.2 times the risk among persons who received ≥2 doses [18]. The adjusted vaccine effectiveness against COVID-19-related hospitalization, ICU admission, and death was 86.3%, 92.2%, and 86.7%, respectively, for a homologous CoronaVac booster [19]. In the fifth COVID-19 wave in Hongkong during early 2022, the exceedingly high mortality rate was attributed to the unvaccinated/not fully elderly. The case fatality rate (CFR) of unvaccinated people was 3.04%, while the CFR was only 0.17% and 0.04% in patients who received two-dose and three-dose vaccination, respectively [20].

In our study, two-dose or three-dose vaccination could shorten the virus clearance time significantly, which is consistent with other studies on viral shedding time of BA.1 variant [21]. Additionally, paxlovid use could shorten the viral shedding time. When paxlovid was initiated within five days after symptom onset, it was associated with an additional reduction in viral load by a factor of 10 as compared with placebo, and it can result in a risk of progression to severe COVID-19 that was 89% lower than the risk with placebo [22]. Treatment with a monoclonal antibody, compared with placebo, was associated with a statistically significant reduction in SARS-CoV-2 viral load [23].

Furthermore, we discovered lymphocyte, CD4^+^T cells, and CD8^+^T cell counts could be served as stratification markers in identifying severe/critical cases. Lymphocytopenia was found to be one of the most common features of COVID-19, especially in CD3^+^T cells [24,25]. Also, cytokines were significantly elevated in severe/critical patients indicating pulmonary inflammation and proinflammatory cytokines are associated with disease severity [11,12,26]. The decrease in CD4^+^ and CD8^+^ T cells indicated T cell dysfunction and immune exhaustion [27]. ACE2 (angiotensin-converting enzyme 2) receptor attaches to cell surfaces in the lung, blood vessels, heart, kidney, and intestines [28]. SRA-CoV-2 can infect cardiomyocytes and cardiac endothelial cells in the heart [29,30]. This study found that cardiac index (D-dimer, LDH, TNT, and BNP) in severe/critical patients was significantly higher than that of mild/moderate patients, and cardiovascular was a significant risk factor for progressing to severe infection in univariate analysis. Although the cardiac index is not routinely tested in infectious diseases, it plays an important role in COVID-19 progression, which guides physicians in anticoagulation treatment and elevates myocardial oxygen. The relationship between severe status and heart damage could be validated in the future.

Several limitations should be mentioned in our study. First, we only enrolled elderly patients from three designated hospitals in Shanghai, and the overall spectrum of COVID-19 infections could not be illustrated. More centres and participants with different health conditions should be included to obtain further scientific analyses. Second, the severe rate could not be deduced in our study, which should be studied in other large-size studies in the future. Third, some data that were missing in laboratory examinations and viral shedding time might introduce bias in data interpretation.

In conclusion, these findings demonstrated that in the elderly aged over 60 years, older age (aged >80), cerebrovascular disease, and chronic kidney disease were associated with an increased risk of severe Omicron infection. Patients with more than two comorbidities were more likely to get a serious outcome. While fully vaccinated/booster could provide strong protection from the severity of COVID-19 among all age groups. It is crucial to emphasize the protection of elders during the COVID-19 pandemic and timely diagnosis and comprehensive treatment of the elderly will also effectively reduce the rate of severe disease progression in this vulnerable group. The limitation of lacking an overall spectrum of COVID-19 infections could be compensated in other larger-scale studies in the future.

## Supplementary Material

Supplemental Material
