# Supplementary material for: Geriatric risk and protective factors for serious COVID-19 outcomes among older adults in Shanghai Omicron wave
Source: Emerg Microbes Infect. 2022 Aug 31;11(1):2045–54. doi: 10.1080/22221751.2022.2109517 (PMC9448390; doi:10.1080/22221751.2022.2109517)
Supplement: Supplemental Material [file TEMI_A_2109517_SM3168.docx]

**Supplementary materials**

**Supplementary Table 1. Influence of comorbidity numbers and vaccination status in different age groups of becoming severe**

**Supplementary Figure 1. The comorbidities in all enrolled patients and severe/critical patients.**

a. The distribution of comorbidity numbers in all enrolled patients.

b. The proportion of different comorbidities in all enrolled patients.

**Supplementary Table 1.**

| Age | Groups | OR | P |
| --- | --- | --- | --- |
| 60-70 years old | comorbidity ≤1 and fully vaccinated/booster | / | 0.996 |
|  | comorbidity ≤1 and unvaccinated/partially vaccinated | 1.196(0.282-5.076) | 0.809 |
|  | comorbidity ≥2 and fully vaccinated/booster | / | 0.998 |
|  | comorbidity ≥2 and unvaccinated/partially vaccinated | **5.357(1.258-22.820)** | **0.023** |
|  |  |  |  |
| 70-80 years old | comorbidity ≤1 and fully vaccinated/booster | / | 0.996 |
|  | comorbidity ≤1 and unvaccinated/partially vaccinated | 0.990(0.326-3.003) | 0.985 |
|  | comorbidity ≥2 and fully vaccinated/booster | 0.684(0.087-5.350) | 0.717 |
|  | comorbidity ≥2 and unvaccinated/partially vaccinated | **3.196(1.088-9.392)** | **0.035** |
|  |  |  |  |
| >80 years old | comorbidity ≤1 and fully vaccinated/booster | / | 0.998 |
|  | comorbidity ≤1 and unvaccinated/partially vaccinated | 0.653(0.334-1.277) | 0.213 |
|  | comorbidity ≥2 and fully vaccinated/booster | 0.835(0.107-6.520) | 0.863 |
|  | comorbidity ≥2 and unvaccinated/partially vaccinated | **1.992(1.025-3.871)** | **0.042** |

**Supplementary Figure 1.**
